# Implementation intensification to disseminate a skills-based caregiver training program: protocol for a type III effectiveness-implementation hybrid trial

**DOI:** 10.1186/s43058-023-00475-7

**Published:** 2023-08-16

**Authors:** Kasey Decosimo, Connor Drake, Cynthia J. Coffman, Nina R. Sperber, Matthew Tucker, Jaime M. Hughes, Leah L. Zullig, Trisha Chadduck, Leah Christensen, Brystana Kaufman, Kelli D. Allen, S. Nicole Hastings, Courtney H. Van Houtven

**Affiliations:** 1 Center of Innovation to Accelerate Discovery and Practice Transformation, Durham VA Health Care System, Durham, NC 27705 USA; 2 Department of Population Health Sciences, Duke University, Durham, NC USA; 3 Department of Biostatistics and Bioinformatics, Duke University Medical Center, Durham, NC USA; 4 Department of Implementation Science, Wake Forest University School of Medicine, Winston-Salem, NC USA; 5 Section On Gerontology and Geriatric Medicine, Division of Internal Medicine, Wake Forest University School of Medicine, Winston-Salem, NC USA; 6 Veteran’s Health Administration Central Office, Washington, DC USA; 7 Duke-Margolis Center for Health Policy, Duke University, Durham, NC USA; 8 Department of Medicine & Thurston Arthritis Research Center, University of North Carolina at Chapel Hill, Chapel Hill, NC USA; 9 Center for the Study of Aging and Human Development, Duke University School of Medicine, Durham, NC USA; 10 Geriatric Research, Education, and Clinical Center, Durham VA Health Care System, Durham, NC USA; 11 Division of Geriatrics, Department of Medicine, Duke University School of Medicine, Durham, NC USA

**Keywords:** Family caregivers, Veterans, Skills training, Informal care, Implementation science

## Abstract

**Background:**

Family caregiver training decreases caregiver psychological burden and improves caregiver depressive symptoms and health-related quality of life. Caregivers FIRST is an evidence-based group skills training curriculum for family caregivers and was announced for national dissemination in partnership with the Veterans Health Administration (VHA) National Caregiver Support Program (CSP). Previous evaluations of Caregivers FIRST implementation highlighted that varying support was needed to successfully implement the program, ranging from minimal technical assistance to intensive assistance and support. However, we do not know the optimal level of support needed to inform cost-effective national scaling of the program. We describe a protocol for randomizing 24 non-adopting VA medical centers 1:1 to a tailored, high-touch implementation support or a standard, low-touch implementation support to test the primary hypothesis that high-touch support increases Caregivers FIRST penetration, fidelity, and adoption. Additionally, we describe the methods for evaluating the effect of Caregivers FIRST participation on Veteran outcomes using a quasi-experimental design and the methods for a business case analysis to examine cost of delivery differences among sites assigned to a low or high-touch implementation support.

**Methods:**

We use a type III hybrid implementation-effectiveness study design enrolling VA medical centers that do not meet Caregivers FIRST adoption benchmarks following the announcement of the program as mandated within the CSP. Eligible medical centers will be randomized to receive a standard low-touch implementation support based on Replicating Effective Programs (REP) only or to an enhanced REP (high-touch) implementation support consisting of facilitation and tailored technical assistance. Implementation outcomes include penetration (primary), fidelity, and adoption at 12 months. Mixed methods will explore sites’ perceptions and experiences of the high-touch intensification strategy. Additional analyses will include a patient-level effectiveness outcome (Veteran days at home and not in an institution) and a business case analysis using staffing and labor cost data.

**Discussion:**

This pragmatic trial will lead to the development and refinement of implementation tools to support VA in spreading and sustaining Caregivers FIRST in the most efficient means possible.

**Trial registration:**

This study was registered on April 8, 2022, at ClinicalTrials.gov (identifier NCT05319535).

**Supplementary Information:**

The online version contains supplementary material available at 10.1186/s43058-023-00475-7.

Contributions to the literature

- This study will illuminate the value of high-touch implementation supports by assessing the extent to which such support improves the penetration of an evidence-based caregiver training program when compared to lower-cost, self-guided  support.
- This study will evaluate the effectiveness of Caregivers FIRST training, thereby informing the field about the benefits to patients of caregiver skills training implemented as a pragmatic trial.
- Assessing the business case of Caregivers FIRST and implementation strategies will inform decision-making related to program scalability and sustainment by informing the VA Health Care System about the costs of delivery using existing clinical teams.

## Background

Family and other unpaid caregivers are the invisible backbone of the US health care system, with approximately 53 million caregivers providing long-term care in the home, including an estimated 5.5 million military caregivers [1, 2]. Education and support to caregivers have been shown to decrease psychological burden, improve caregiver depressive symptoms, and enhance health-related quality of life for caregivers [3–6]. The 2022 National Strategy to Support Family Caregivers highlights the need to strengthen services and supports for family caregivers, where less than 10% of caregivers report that they receive the training they need to fulfill their caregiving role [7], and only 30% use supportive services [8]. One means of assisting individuals engaged in caregiving is to expand training for them [9]. The National Academies of Science, Engineering and Medicine published a landmark report recommending the spread of evidence-based trainings for caregivers as a means of expanding support [10].

The most comprehensive publicly funded caregiver support program in the country is led by the US Department of Veterans Affairs. The VA Caregiver Support Program (CSP) provides one-on-one coaching and support, group support, skills training, respite care, peer mentoring, counseling, and connection to resources for caregivers of Veterans across the continuum of care [11–13]. Over the past 10 years, CSP has promoted a culture of innovation by tapping input from caregivers and field staff to develop new supports including peer support groups, intimate partner violence trainings, and diagnosis-specific trainings for caregivers on dementia and post-traumatic stress disorder [11]. Caregivers FIRST is an evidence-based program embedded in the Optimizing Function and Independence Quality Enhancement Research Initiative (Function QUERI) which promotes function and independence in older Veterans and their family caregivers [14]. In 2018, CSP partnered with Function QUERI to implement Caregivers FIRST in eight VA medical centers, in part because CSP identified a gap in group caregiver trainings available and because results from a single-site randomized control trial of Caregivers FIRST improved caregiver and Veteran experiences of VA health care [15–17]. From 2018 to 2020, Caregivers FIRST was successfully implemented at those eight VA medical centers. Implementation support was guided by the Replicating Effective Programs (REP) framework and included five facilitated calls with each site, a two-day site visit, and technical assistance [15]. Based on the demand for caregiver training and high program satisfaction from caregivers [16], CSP announced Caregivers FIRST for national dissemination as a ‘strong practice’ program, meaning facilities could elect to begin offering in fiscal year 2021 [18] and then a mandated ‘minimum standard’ program, where all sites must begin offering in fiscal year 2022.

Few evidence-based caregiver support interventions have been scaled for widespread dissemination in the USA. One approach to addressing implementation barriers is to test the impact of implementation support strategies on the reach and integration of new practices into existing systems of care [10]. With Caregivers FIRST implementation, we observed barriers across multiple levels with the initial eight VA medical centers. For example, at the team level, Caregivers FIRST required novel practice patterns and coordination across service lines. At the provider level, successful implementation required time-intensive training and technical assistance (approximately 100–140 h per site). Furthermore, the level of support participating medical centers needed to successfully integrate Caregivers FIRST into routine practice significantly varied. Given competing clinical responsibilities for members of the implementation team and the costly nature of time-intensive approaches to enhance implementation delivered by research teams, it is essential to minimize the time and resources needed to implement. However, we do not know the optimal level of support needed to successfully offer Caregivers FIRST in diverse clinical contexts as part of routine care patterns, which is vital to inform cost-effective national scaling of the program. Supporting the adoption and implementation of Caregivers FIRST in routine care settings nationally requires the design and evaluation of promising and scalable implementation support strategies that leverage implementation facilitators and overcome identified barriers.

We describe a protocol for a type III effectiveness-implementation hybrid trial to evaluate the effectiveness of high-touch implementation support on Caregivers FIRST penetration, fidelity, and adoption implementation outcomes compared to low-touch implementation support in medical centers that did not adopt the program following the VA Health Care System announcement that Caregivers FIRST could be delivered to meet expected performance standards. The hybrid design was selected to evaluate both implementation and effectiveness outcomes and was informed by the goals of the nationwide VA Health Care System to require 142 medical centers to provide at least two caregiver group trainings in a fiscal year to meet Caregiver Support Program minimum standards. Partnered with the CSP, this project’s aims are three-fold. First, and the primary goal of this project is to evaluate the effectiveness of implementation support on implementation outcomes at 12 months in a cluster randomized trial (Aim 1). Second, in exploratory analysis, we will assess the effectiveness of Caregivers FIRST to increase Veteran patient’s time at home over 6 months, compared to similar patients whose caregivers did not participate in Caregivers FIRST (Aim 2). Third, we will conduct a business case analysis of the high- and low-intensity implementation support to identify cost-efficient strategies for facilitating the spread of Caregivers FIRST (Aim 3). Mixed methods best practices will be used to obtain qualitative data to better understand the sites’ experience with Caregivers FIRST and the implementation strategies offered. In addition to filling gaps in evidence surrounding implementation strategies needed to scale caregiver programs, this pragmatic trial will lead to the development and refinement of implementation tools to support VA in spreading and sustaining Caregivers FIRST in the most efficient means possible.

## Methods

### Overview

**Fig. 1:**
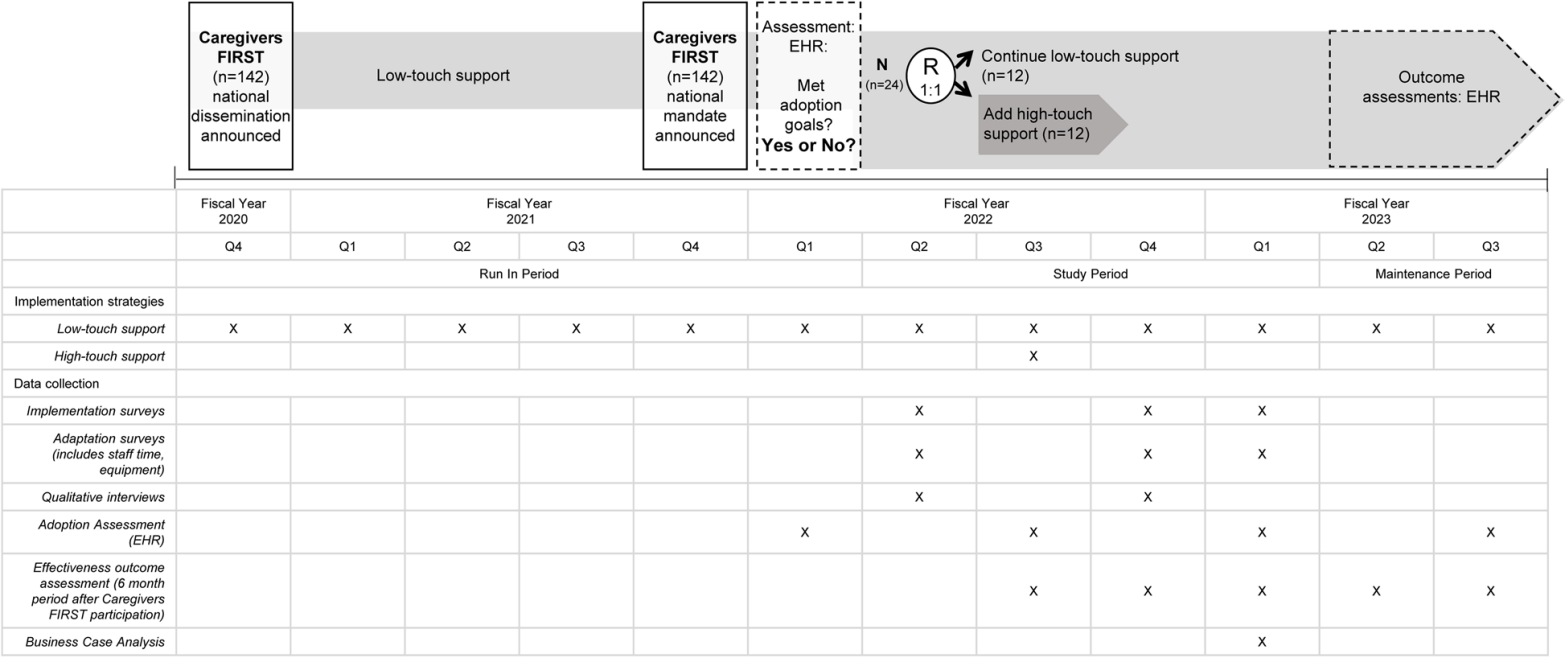
Study schema by fiscal year quarter

We hypothesize that high-touch implementation support will directly influence delivery teams’ capacity and skills to effectively self-organize and problem-solve and will lead to higher implementation penetration, fidelity, and adoption to Caregivers FIRST compared to foundational support (low-touch). We will use a cluster randomized trial to randomly assign medical centers 1:1 to either (i) foundational “low-touch” implementation support (active comparator) or (ii) enhanced “high-touch”  support (experimental) (Fig. 1). The Standards for Reporting Implementation Studies (StaRI) checklist is available as supplemental material.

### Randomization

A stratified block randomization will be used with two dichotomous stratification variables (1) site complexity (low complexity (2, 3 and no complexity) versus all others) [19] and (2) prior implementation of Caregivers FIRST (implementation in FY21 or FY22 vs. none). Clinical delivery staff will not be blinded to their randomization track nor will any Function QUERI study staff.

### Caregivers FIRST intervention

Caregivers Finding Important Resources, Support, and Training (FIRST) is an evidence-based group skills training for friends or family members of Veterans and is designed to be delivered by existing clinical staff. Core and adaptable components of the Caregivers FIRST intervention were developed in prior work and refined over time using caregiver, provider, and operational partner input [15, 16, 20, 21], making its design user-centered. VA medical centers implementing Caregivers FIRST must deliver the core components which include four sessions that address caregiver coping, support seeking, hands-on, and health system navigation skills [15, 21]. Allowing for alignment with resources available at each VA medical center, adaptable components include the context or service line Caregivers FIRST is offered whether it be within the CSP or in partnership with multiple VA service lines (e.g., chaplaincy, mental health, primary care). Similarly, the approved adaptations allow flexibility with delivery pace (e.g., weekly or consolidated over fewer days) and modality (e.g., in-person, phone, video conference) [21]. Sites can decide on their own target caregiver population to recruit (e.g., based on needs in a given service line or caregivers of a specific diagnosis), but are told that Caregivers FIRST was developed for caregivers of Veterans with functional impairment and not based on a single diagnosis. Eligible caregivers who participate in Caregivers FIRST are enrolled in either the VA CSP’s Program of General Caregiver Support Services (PGCSS) or the Program of Comprehensive Assistance for Family Caregivers (PCAFC).

### Site inclusion criteria

Site eligibility for this study will be VA medical centers that do not adopt Caregivers FIRST defined as sites that had no Caregiver FIRST activity or sites that implemented the program but had less than five unique caregivers trained in the first 6 months of the mandated period (October 1, 2021–March 31, 2022). The threshold of less than five caregivers trained was determined in partnership with CSP as “not adopting.” Adoption metrics will be tracked using a dashboard developed jointly with CSP to monitor site implementation, given individual sites are required to document the delivery of Caregivers FIRST and attendance. Ineligible sites will include the eight VA medical centers that had previously participated in Function QUERI, since in a previous trial, those sites were exposed to a high-touch implementation strategy [15]. Eligible sites will be identified by the dashboard and will be prioritized by VA service region, site complexity level, implementation activity (e.g., did not adopt versus implemented but with “low enrollment” defined as less than five caregivers), and CSP staffing capacity (CSP total staffing FY20). The goal will be to select from medical centers distributed across VA service regions (approximately two per region) with sites systematically selected to approach for enrollment based on complexity level and lowest implementation activity within a VA region. Additional inclusion criteria include (1) facility leadership willing to participate in the study via a signed participation agreement and (2) agreement to deliver Caregivers FIRST within 6 months.

### Implementation framework

**Fig. 2:**
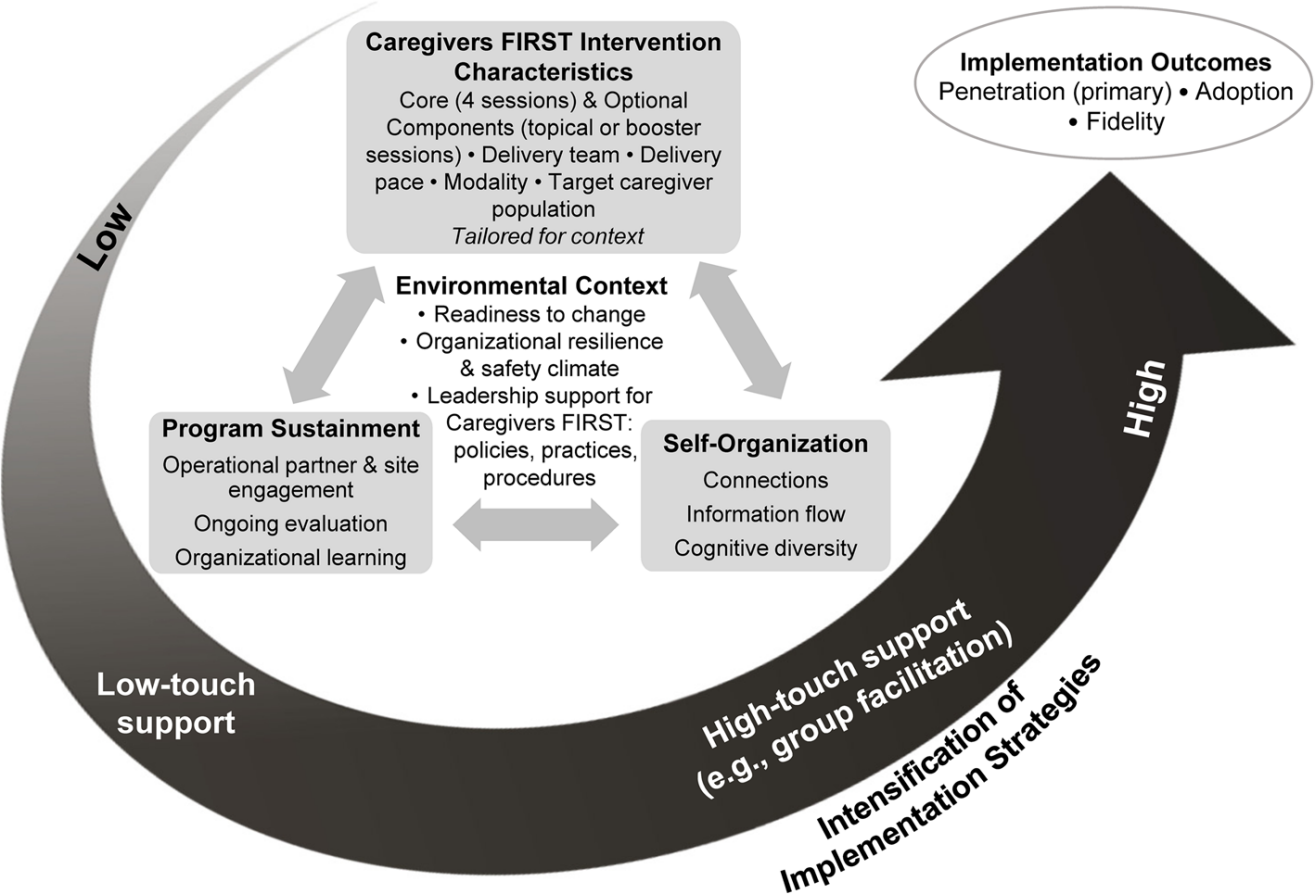
Function QUERI implementation intensification framework

Understanding implementation as a dynamic and iterative process, we will use an overarching framework guided by the QUERI Implementation Roadmap and the Dynamic Sustainability Framework [14, 22, 23]. In this framework, implementation outcomes of new programs are influenced by the characteristics of the intervention and the interaction of factors within a site’s environmental context and processes to support program sustainment, as well as the delivery team’s capacity to self-organize for optimal problem-solving [14]. Because of variations in context and organizational characteristics, we propose that foundational (low-touch) implementation support will be sufficient for many VA medical centers to be able to successfully incorporate Caregivers FIRST into routine practice. For the enrolled sites with low adoption, we hypothesize that enhanced (high-touch) implementation support will influence readiness to change, organizational resilience and safety climate, and leadership support which will lead to higher Caregivers FIRST penetration, fidelity, and adoption (Fig. 2).

### Aim 1: implementation strategies

Among the enrolled Caregivers FIRST sites, the goal is to test implementation intensification approaches, specifically foundational (low-touch) vs. enhanced (high-touch) implementation support, informed by Replicating Effective Programs (REP). REP is a framework ideal to support the delivery of interventions in new settings or to new populations [24, 25], and it has been pragmatically applied in implementation studies in the VA [15, 26, 27]. REP includes four phases: pre-conditions, pre-implementation, implementation, and maintenance/evolution, with emphasis on local customization and tailoring. We anticipate low-touch implementation support, which provides self-guided tools to be used by a variety of site settings with varying levels of resources and experience with quality improvement or innovation, will be sufficient to successfully incorporate Caregivers FIRST into routine practice.

#### Foundational implementation support (low-touch)

All enrolled sites will receive foundational support, a bundle of self-guided implementation strategies that include toolkits for implementation. The low-touch strategy includes four support components that were developed and tested in prior Function QUERI work [15]: toolkit; SharePoint access for clinical program training materials; data dashboard to assist sites with tracking their own data; and learning collaboratives to promote peer-to-peer sharing and implementation support (Table 1).

**Table 1:** Components of low-touch and high-touch implementation support

Package	Component	Description
**Foundational (low-touch)**	Toolkit	Standardized program materials and training curriculum to help clinical staff implement Caregivers FIRST, including recorded implementation support training webinars and guidance on core and modifiable components, including options for customization
	SharePoint	Secure SharePoint for access to Caregivers FIRST curriculum, implementation support materials (e.g., caregiver marketing templates), and standardized materials to facilitate monitoring sites’ progress
	Data dashboard	Secure web-based dashboard developed in partnership with the Caregiver Support Program to assist sites with tracking their implementation activity through electronic health record notes
	Learning collaboratives	Designed to capture and share local knowledge as well as create a collaborative environment for peer-to-peer sharing of experiences and best practices to support implementation. Delivered via teleconference as monthly office hours and quarterly Diffusion Network calls
**Enhanced (high-touch)**	Foundational low-touch support	All sites randomized to high-touch support also receive standard low-touch support
	Technical assistance	Direct access to Function QUERI implementation facilitators for tailored technical assistance as needed
	Needs Assessment	Online Needs Assessment (survey) to ensure calls address key implementation challenges
	Facilitated calls	Four groups facilitated calls focusing on key barriers to implementation and needs identified from the Needs Assessment

#### Enhanced implementation support (high-touch)

Sites randomized to receive high-touch support will receive four facilitated phone calls over a period of approximately three months that address adoption barriers and elicit sharing of successful adoption strategies. The high-touch support will consist of facilitation, a process of interactive problem-solving and support that occurs in a context of a supportive interpersonal relationship. The study team plans to conduct four scheduled calls over three months in a group format with sites randomized to high-touch support, providing tailored facilitation around key barriers to Caregivers FIRST implementation and program sustainment (Table 1). Facilitation will be provided by Function QUERI team members, comprised of investigator and masters-level project staff who have received QUERI implementation facilitation training [28]. At the end of each call, study team facilitators will summarize action plans for the purpose of sites to have tangible steps to complete in between calls.

### Aim 1: data collection and measures

Caregivers FIRST implementation will be evaluated using an explanatory sequential mixed method design where qualitative data will be collected in parallel with quantitative data to explore and understand contextual and experiential factors related to differences in site-level implementation outcomes [29]. Evaluation data sources will be collected through (1) CSP program data for implementation outcomes (Aim 1), (2) quantitative surveys (baseline, 6 months, and 12 months), (3) semi-structured interviews (sample of sites at baseline and 6 months), and (4) surveys of program adaptations. For additional context for Aim 1, other data sources include (5) electronic health record data for effectiveness outcomes/quality metrics and (6) staff and labor cost data to assess the business case delineated by program delivery and implementation strategy. Evaluation data sources, methods of data collection, source, and timepoint are summarized in Table 2. Surveys and qualitative interviews were approved as exempt research by the Institutional Review Board of the Durham VA Health Care System and received concurrence from the VA national labor unions.

**Table 2:** Study measures by aim

	Construct	Measure	Data source	Timepoint(s)
Aim 1: implementation	Penetration (primary)	Proportion of caregivers who received consults for VA caregiver education and training services who attended at least one class	EHR data	12 months
		Number of classes delivered by site	EHR data	12 months
	Adoption	Minimum of four training classes delivered to at least five caregivers	EHR data	12 months
	Fidelity	Proportion of number of recommended classes delivered by site	EHR data	12 months
		Number of caregivers attended at least one class by site	EHR data	12 months
		Mean number of classes attended per caregiver	EHR data	12 months
Aim 2: effectiveness	Quality metric	Inclusion of Caregivers FIRST in VA medical center performance plans	VA Network Director and Facility Director Performance Plan	12 month fiscal year
		Number of caregivers enrolled in the Program of General Caregiver Support Services (PGCSS)	EHR data	12 months
	Effectiveness outcome	Veteran days in the community (e.g., not in a hospital or nursing home) after caregiver participation in Caregivers FIRST	EHR data	6 months
Aim 3: cost	Delivery costs	Number of hours staff spent delivering one round of Caregivers FIRST training	Survey	Baseline, 6 months, 12 months
	Implementation costs	Number of hours staff spent participating in implementation strategies	Survey Attendance logs Meeting notes	Baseline, 6 months, 12 months
Contextual and experimental factors	Implementation climate	Leadership and Motivation to Implement	Survey Qualitative interviews	Baseline
		Implementation Climate Scale [30]	Survey	Baseline
		Organizational Resilience [31]	Survey	Baseline, 6 months, 12 months
		Challenging and Helpful Implementation Factors	Survey Qualitative interviews	Baseline, 6 months, 12 months
	Readiness for implementation	Organizational Readiness for Implementing Change [32]	Survey	Baseline, 6 months, 12 months
	Adaptations	Framework for Reporting Adaptions and Modifications Expanded (FRAME) [33]	Survey	Baseline, 6 months, 12 months
	Process	Implementation Process and Experience	Survey Qualitative interviews	6 months, 12 months
	Policy and incentives	Program Sustainability Index [34]	Survey	6 months, 12 months

#### Aim 1: implementation outcomes

Implementation outcomes informed by Proctor and colleagues [35] will be assessed through electronic health record (EHR) data pulls from the VA Corporate Data Warehouse. Prior to the announcement of Caregivers FIRST as a mandated CSP training, and in consultation with Function QUERI, CSP developed and launched a national EHR template for clinical staff to document attendance and details of Caregivers FIRST participation in individual caregiver’s VA electronic health records. Data for implementation outcomes will be extracted from health factors that are generated from the national EHR templated note. The primary outcomes are cluster-level PENETRATION defined as the (1) proportion of caregivers who received consults for VA caregiver education and training services who attended at least one class at a medical center over a 12-month period and (2) the number of classes delivered over a 12-month period. FIDELITY outcomes are cluster-level defined as (1) the proportion of the number of recommended classes delivered over a 12-month period, (2) the number of caregivers who attended at least one class over a 12-month period, and (3) the mean number of classes attended per caregiver over a 12-month period. ADOPTION is a cluster-level dichotomous outcome defined as meeting a threshold of four or more training classes delivered to a minimum of five caregivers over a cumulative 12-month period (yes) or not meeting this threshold is considered non-adoption (no). In addition, while 12 months is the primary endpoint, penetration and fidelity outcomes will also be assessed over 6- and 18-month time periods with descriptive results.

#### Quantitative staff surveys

Quantitative surveys will be administered using VA REDCap (Research Electronic Data Capture) [36] to all staff identified as delivery team members or relevant service line leaders by the site Point of Contact for each enrolled site. An initial invitation will be emailed, with two follow-up weekly reminders. Factors influencing implementation that were highlighted in our overarching framework (characteristics of the intervention, site’s environmental context, and delivery team’s capacity to self-organize) will be captured with baseline survey measures including organizational resilience [31], organizational readiness [32, 37], and implementation climate [30]. The 6- and 12-month survey measures include program sustainability [34], organizational resilience [31], and experience with implementation strategies. For both baseline and 12-month surveys, we will also collect information on challenging and helpful implementation factors with categories developed from previous Function QUERI implementation work.

#### Qualitative staff interviews

With a sample of half of the enrolled sites stratified by arm, we will conduct 30-min individual telephone interviews with Caregivers FIRST delivery staff and service line leaders (identified by each site’s Point of Contact) to elicit facilitators and barriers that affected implementation. A semi-structured interview guide will probe based on Proctor’s criteria for specifying and reporting strategies (e.g., actor, action, target, temporality, frequency) [38] as well as conditions relevant for implementing Caregivers FIRST. We will use maximum variation sampling to ensure diversity by implementation status (e.g., adopted vs. did not adopt or had “low enrollment”) and size of Caregivers FIRST delivery team. Participants will be invited via email, and we will aim for 5–10 interviews per sampled SITE. Baseline interviews will assess implementation climate, readiness, and available resources and 6-month interviews will assess experience with implementation strategies and tools, including those who received high-touch support, and any additional strategies developed during program implementation. All interviews will be audio recorded and transcribed.

#### Program adaptations

Points of Contact from all enrolled sites will also be asked to report Caregivers FIRST adaptations via the VA REDCap survey at baseline, 6 months, and 12 months. An initial invitation will be emailed, with two follow-up weekly reminders. Survey questions are derived from Wiltsey Stirman’s Framework for Reporting Adaptions and Modifications Expanded (FRAME), which provides a framework for standardized tracking of modifications and monitoring of Caregivers FIRST fit within each site [33]. Examples include when the modification was made, whether the adaptation was made proactively, and key decision-makers. Example modifications to probe upon are based upon prior work [21], including the use of optional components, delivery format, delivery team composition, and additions or removal of content. The survey will also ask about equipment and staff time spent in planning and delivery to assess cost.

### Aim 2: effectiveness outcomes/quality metrics measures

Effectiveness outcomes will include Veteran days in the community (e.g., not in a hospital or nursing home) over a 6 month period after caregiver participation in Caregivers FIRST. We will use the inclusion of Caregivers FIRST in VA medical center performance plans as the quality metric, where sites report the status on meeting requirements within the CSP Program of General Caregiver Support Services (PGCSS). In addition, we will evaluate the number of caregivers enrolled with each site’s PGCSS and whether the quality metrics change based on the implementation approach.

### Aim 3: analyses of business case/value proposition measures

The business case will include a budget impact analysis to help VA administrators consider the cost of implementation and delivery along with the effectiveness of the intervention in the context of the VA priorities to improve Veteran outcomes. The budget impact analysis will evaluate implementation costs for each strategy and intervention delivery costs per participant. The high-touch implementation support strategy is expected to use more resources (delivery time and attendance time) and potentially improve implementation outcomes, for example improved fidelity or increased penetration. To assess the value of the additional investment in implementation support, we will describe implementation costs per enrolled participant for each implementation strategy. While the overall resource use might be higher, a lower cost per participant would indicate a high value. We will use staff time and equipment data from the adaptations survey (baseline, 6 months, and 12 months) to assess costs using U.S. Veterans Health Administration salary and managerial cost accounting data. Drawing from methods in prior Function QUERI work [17], Business Case Analysis (BCA) will frame affordability to VA. For the cumulative 12-month study period, two types of costs will be collected for each site using a standardized method to track implementation activities 1) clinical delivery team costs incurred and 2) implementation strategy costs incurred. Because the 24 sites enrolled will represent a fraction of the sites nationally who will also implement (expect over 100 to implement), we can extrapolate to a national scenario in the BCA.

## Analysis

### Aim 1: implementation of quantitative analysis

As part of our type III effectiveness-implementation hybrid design framework, the primary research question compares differences in implementation outcomes (penetration, fidelity, and adoption) between implementation arms at 12 months. Implementation outcomes are continuous, count, and binary outcomes, and generalized linear models [39] will be used to examine the effect of high-touch support on implementation outcomes at 12 months. The main predictor of interest will be enhanced (high touch) implementation support vs. foundational (low touch) implementation support, and stratification variables for site complexity (low complexity (2, 3, and no complexity) versus all others) and prior implementation of Caregivers FIRST or not will be included; other facility level covariates for consideration are CSP program quality score and size of CSP program. In secondary analyses, implementation outcomes over a 6-month and 18-month period will be assessed and described. We will examine how implementation outcomes change over time using descriptive methods (e.g., plots, descriptive statistics, subgroups).

Descriptive statistics for survey measures such as organizational readiness for implementation change (see Table 2 for others) will be calculated overall and by randomization arm. We will use the same modeling approach described above to examine the effect of the implementation strategy on survey measures.

### Aim 1: qualitative analysis and mixed methods

Interview transcripts will be coded and analyzed by at least two Function QUERI team members. Using Expert Recommendations for Implementing Change (ERIC) typology [40] and clusters [41], we will use directed content analysis including a priori labels to mark implementation strategies and activities and data-derived labels to reflect respondents’ description of their experience with barriers to implementation. A framework matrix will be developed to summarize coded data and compare implementation context, experience, and barriers across sites. Matrix rows will reflect coded implementation support types and the columns will reflect whether or not responses are from low-touch sites and implementation outcomes for each site. Summaries of coded data within each matrix cell will describe the implementation strategy.

### Aim 2: effectiveness outcomes

We will assess the impact of Caregivers FIRST on Veteran days in the community (home time) over a 6-month period. Our sample of Veterans for this analysis will come from sites that adopt Caregivers FIRST nationally (that is, not limited to the 24 enrolled sites). The sample of Veterans will be from those whose caregiver received consults for VA caregiver education and training services, including to Caregivers FIRST over a year period after the program was announced as a minimum standard; some of those caregivers will attend Caregivers FIRST classes (i.e., treatment group) and some will not (i.e., comparison group). We will use inverse-probability of treatment weighted methods to adjust for confounding and estimate an average causal treatment effect for Caregivers FIRST on home time over the 6-month time period following the consult for a VA caregiver education and training. The study team will determine a priori which covariates are likely to be key determinants of attending Caregivers FIRST training. We will explore the distribution that best fits the days at home outcome: Poisson, Negative Binomial, or zero-inflated. Generalized linear models with inverse propensity score weights will be used. We will describe the quality metrics using descriptive statistics, but these will not be modeled.

### Aim 3: business case analysis

The base-case BCA will use a decision tree to compare the expected value for costs (implementation and delivery) and implementation outcomes (number of participants) between the two implementation support arms. In addition, we will use one-way and probabilistic sensitivity analysis to simulate likely outcomes in the context of distributions informed by the trial data as well as prior evidence. By modeling plausible scenarios, the decision model will allow us to communicate a practical range of estimates to sites and operational partners rather than relying on statistical significance. We will work with CSP to establish thresholds.^56^ We will report costs by arm for the base case BCA and for different assumptions, along with what conditions would need to be met to be within VA thresholds for affordability/value.

### Sample size and power

Sample size calculations were conducted for the penetration implementation outcomes at 12 months. Based on a two-sided t-test based on a sample size of 24 sites (12 receiving foundational low-touch implementation supports and 12 receiving enhanced high-touch support), a type-1 error rate of 5%, will have 80% power to detect an effect size difference of 1.2 and 90% power to detect an effect size difference of 1.4 between arms. We did not adjust alpha for co-primary outcomes as success will be assessed based on improvement in both measurements of penetration (Table 2).

## Discussion

Health care systems need resource-efficient approaches to scale caregiver training interventions. This study is expected to illuminate the value of a high-touch implementation strategy to promote the penetration or integration of Caregivers FIRST within routine practice. The mixed methods design will help identify the right dose of implementation support for the efficient scaling of future caregiver training programs. Identifying the ‘right dose’ of implementation support to successfully implement is an important gap currently in the research literature because very few research-driven caregiver programs are disseminated after the research project concludes. In particular, we do not have benchmarks on expected rates of reach from most caregiver support and training studies, and as such, have little ability with which to frame their success as scaled pragmatic trials. In the first rendition of Caregivers FIRST at eight VA medical centers, we reached 29% of eligible caregivers with our training. And yet, we do not have other evidence to tell us whether that was a high or low rate of reach. The rates of penetration, fidelity, and adoption discovered in this study could contribute to developing a range of benchmarks as other teams scale their caregiver interventions in health systems.

In addition, given the VA Caregiver Support Program is in the practice of implementing new programs annually as a part of its mission, our findings could be instructive to them and to other operations decisions in health systems. For example, learning that low-touch implementation support works just as well for sites as a high-touch approach could reduce the barriers to health systems to initiate caregiver training supports. Alternatively, identifying which types of sites might benefit from a high-touch approach could help health systems target scarce resources to implement caregiver trainings. In general, this study should provide real-world evidence of the effort needed to implement caregiver trainings, which is timely as the National Caregiver Strategy to Support Family Caregivers recommended in 2022 spreading evidence-based trainings as a tenet of its recommendations.

Results from Aim 2 will help build the evidence of how pragmatic trials affect patient outcomes. Revisiting the effectiveness of Caregivers FIRST training in its current pragmatic trial form can inform the benefits to patients of caregiver skills training. Finally, assessing the business case of Caregivers FIRST in Aim 3 will inform the future scalability and sustainment of the program by informing the VA Health Care System about the costs of delivery using existing clinical teams.

### Limitations

Our study is expected to have limitations. Assessment of implementation outcomes will rely on EHR data being entered appropriately in EHR templates by clinical staff. As such, we will rely on mixed methods to fully assess the primary aim of this work to assess fully the value of high-touch over low-touch implementation supports. Obtaining the perspectives from program delivery staff will add richness to our data and help us understand the types of sites who may benefit from low versus high-touch implementation supports. Missing data from sites would be an additional limitation. Regarding business case analysis, we will be using the VA perspective and considering implementation in an integrated health care system. Sites will report time spent implementing the program, which could also be a limitation if data are not completely reported. Additionally, costs of implementation will be primarily labor costs, but it is unknown how business case analysis findings would generalize to private or non-integrated health care systems.

We feel the strengths, described above, outweigh the anticipated limitations. Importantly, we use a partner-informed and user-centered design that incorporates feedback and the needs of both caregivers, clinical delivery teams implementing Caregivers FIRST, and our operational partners in the VA Caregiver Support Program. Since its inception as a single-site randomized control trial in 2014, the Caregivers FIRST curriculum has been improved collaboratively with sites, using input from multiple disciplines and assuring high-quality content and better cultural competence. Sites have also been very willing to engage in surveys and interviews with our team, leading to rich data collection and impressions of their experiences.

### Supplementary Information

**Additional file 1.** Standards for Reporting Implementation Studies: the StaRI checklist for completion.

## Data Availability

Not applicable.

## Abbreviations

BCA: Business Case Analysis
Caregivers FIRST: Caregivers Finding Important Resources, Support, and Training
CGF: Caregivers FIRST
CSP: Caregiver Support Program
ERIC: Expert Recommendations for Implementing Change
EHR: Electronic Health Record
FRAME: Framework for Reporting Adaptions and Modifications Expanded
Function QUERI: Function and Independence Quality Enhancement Research Initiative
PCAFC: Program of Comprehensive Assistance for Family Caregivers
PGCSS: Program of General Caregiver Support Services
QUERI: Quality Enhancement Research Initiative
REP: Replicating Effective Programs
VA: Veterans Affairs
VHA: Veteran Health Administration
VAMC: VA medical center
